# Analyzing Trends in Demographic, Laboratory, Imaging, and Clinical Outcomes of ICU-Hospitalized COVID-19 Patients

**DOI:** 10.1155/2023/3081660

**Published:** 2023-05-29

**Authors:** Mohsen Gholinataj Jelodar, Shahab Rafieian, Azadeh Allah Dini, Fatemeh Khalaj, Samira Zare, Hanieh Dehghanpour, Samaneh Mirzaei

**Affiliations:** ^1^Department of Internal Medicine, Shahid Sadoughi University of Medical Sciences, Yazd, Iran; ^2^Clinical Research Development Center, Shahid Rahnemoon Hospital, Shahid Sadoughi University of Medical Sciences, Yazd, Iran; ^3^Department of Thoracic Surgery, Imam Khomeini Hospital, Tehran University of Medical Sciences, Tehran, Iran; ^4^Department of Health in Emergencies and Disasters, School of Public Health, Shahid Sadoughi University of Medical Sciences, Yazd, Iran

## Abstract

**Background:**

COVID-19 has led to significant hospitalization and intensive care unit admission rates. The demographic parameters of COVID-19 patients, such as age, underlying illnesses, and clinical symptoms, substantially influence the incidence and mortality of these individuals. The current study examined the clinical and demographic characteristics of COVID-19 intensive care unit (ICU) patients in Yazd, Iran.

**Methods:**

The descriptive-analytical cross-sectional study was conducted on ICU patients with a positive RT-PCR test for coronavirus, admitted to the ICU in Yazd province, Iran, over 18 months. To this end, demographic, clinical, laboratory, and imaging data were collected. Moreover, patients were divided into good and worse clinical outcome groups based on their clinical outcomes. Subsequently, data analysis was performed at a 95% confidence interval (CI) using SPSS 26 software.

**Results:**

391 patients with positive PCR were analyzed. The average age of the patients in the study was 63.59 ± 17.76, where 57.3% were male. On the high-resolution computed tomography (HRCT) scan, the mean lung involvement score was 14.03 ± 6.04, where alveolar consolidation (34%) and ground-glass opacity (25.6%) were the most prevalent type of lung involvement. The most common underlying illnesses in the study participants were hypertension (HTN) (41.4%), diabetes mellitus (DM) (39.9%), ischemic heart disease (IHD) (21%), and chronic kidney disease (CKD) (20.7%). In hospitalized patients, the rates of endotracheal intubation and mortality were 38.9% and 38.1%, respectively. Age, DM, HTN, dyslipidemia, CKD, cerebral vascular accident (CVA), cerebral hemorrhage, and cancer were reported to be significantly different between these two groups of patients, indicating an increase in the rate of intubation and mortality among these patients. Furthermore, the multivariate logistic regression analysis revealed that DM, HTN, CKD, CVA, neutrophil-to-lymphocyte ratio (NLR), the percentage of lung involvement, and initial O_2_ saturation significantly increase the mortality of ICU patients.

**Conclusion:**

Several features of COVID-19 patients influence the mortality in these individuals. According to the findings, early detection of this disease in people at high risk of death can prevent its progression and lower mortality rates.

## 1. Introduction

Coronavirus disease 2019 (COVID-19) has resulted in many hospitalizations and ICU admissions [1]. Although this condition is typically mild and asymptomatic, it can cause severe pneumonia necessitating ICU hospitalization [2], which is one of the leading causes of mortality worldwide. Various studies have revealed that the rate of ICU hospitalization after this disease ranges from 5 to 32% [3], and the mortality rate ranges from 30.9% to 97.2% [2, 4–7]. Moreover, the reported death rate for individuals requiring a mechanical ventilator ranges from 50 to 97%. This frequency was considerably higher than the recorded mortality rates of 35% to 46% for patients with H1N1 influenza pneumonia and other types of acute respiratory distress syndrome (ARDS) [8]. The mortality rate attributable to this virus was estimated to be 3.4% in a study [9]. Different age groups experience varying rates of mortality. In the United States, the death rate ranges from 10 to 27% for those over 85 years old, 3 to 11% for those 65 to 84 years old, 1 to 3% for those 64 to 55 years old, and less than 1% for those aged between 20 to 54 years old [10].

COVID-19 is diagnosed using laboratory, clinical, and diagnostic imaging data [11]. Furthermore, reverse transcriptase-polymerase chain reaction (RT-PCR) was introduced as a standard diagnostic method for identifying viral particles. A chest CT scan's sensitivity for identifying COVID-19 varies based on the stage of the disease [12]. In early COVID-19 diagnosis, radiological examinations are essential. Ground glass opacity (GGO) is the most common result on chest CT scans and occurs in most individuals with bilateral lung involvement [13].

Coronavirus is most often related to cardiovascular diseases and HTN, followed by diabetes DM [14]. COVID-19 patients are at an increased risk of mortality and severity as their age and comorbidities (such as cardiovascular disease, DM, chronic lung disease, and HTN) increase. However, the male death rate is 2.4 times that of females of the same age (70.3% vs. 29.7%, respectively) [15]. Studies have demonstrated that COVID-19 is particularly likely to affect older males with comorbidities and is associated with acute respiratory distress syndrome (ARDS) [16]. Older patients with chronic conditions are at greater risk for organ failures, such as shock, ARDS, IHD, and CKD, and thus have a higher mortality rate than younger and middle-aged patients with COVID-19 [17].

Information on the characteristics and clinical outcomes of individuals with severe COVID-19 is limited; however, reducing mortality is essential [18]. The incidence and mortality of COVID-19 individuals are highly influenced by the demographic features of these patients, such as age, underlying diseases, and symptoms. Investigating the characteristics of COVID-19 patients will aid in forming an accurate picture of the patient's state and facilitating the provision of superior medical care. In addition, these results have significant implications for a better understanding of the epidemiology of COVID-19 and the improved planning and organization of hospitals and critical care units to guarantee the readiness and optimization of care delivery in pandemic circumstances.

According to the literature review, no long-term study on ICU patients has been conducted on the clinical findings and imaging of COVID-19 cases. Consequently, the current study aimed to show 18 months of research on the ICU patients of Yazd Province in Iran. Notably, Yazd province has the most significant rate of involvement with diabetes mellitus in Iran [19]. Since previous research identified diabetes as the most crucial risk and severity factor of COVID-19, conducting the current study seemed essential. Therefore, the present investigation aims to explore the clinical and demographic aspects and outcomes associated with COVID-19 in patients.

## 2. Material and Methods

### 2.1. Study Design and Setting

This research is a descriptive-analytical cross-sectional study. The study population consisted of patients with a positive RT-PCR test for coronavirus, admitted to the ICU in Yazd province, Iran, from March 20, 2020, to September 1, 2021. The study population comprised 391 patients admitted to the ICU with a positive RT-PCR test for coronavirus. This study was assigned the code IR.SSU.SRH.REC.1400.011 by the ethical committee of Yazd University of Medical Sciences.

### 2.2. Data Collection and Outcome

ICU patient medical records were utilized for data collection. To this end, the patient's demographic, clinical, laboratory, and imaging data were collected. Patient data included demographic information (age, gender, comorbidity disease), clinical information (duration of hospital stay, duration of hospitalization in ICU, arterial blood oxygen saturation (O_2_ sat) upon arrival and discharge in ICU), disease-related complications (noninvasive ventilation (NIV), intubation, death, gastrointestinal bleeding, venous thrombosis, secondary infection), laboratory data (lactate dehydrogenase (LDH), serum creatinine phosphokinase (CPK), creatinine (Cr), urea, neutrophil lymphocyte ratio (NLR), white blood count (WBC), lymphocytes), and imaging (percentage of lung involvement determined through high-resolution computed tomography (HRCT) scan).

CT scan involvement was evaluated based on CT scan observation and the radiologist's assessment of the severity and type of lung involvement. The severity of lung involvement was determined by rating the percentage of lung involvement in each lobe, with the ultimate score being based on the sum of the lobe scores. The lung involvement score for each lobe was computed according to the Fleischner Society's glossary-defined scoring system as follows: no lung involvement 0, 5% involvement: 1, 5 to 25%: 2, 25 to 50%: 3, 50 to 75%: 4, and more than 75% involvement: 5. Consequently, lung involvement was assigned a score between 0 and 25 based on this scoring method. The overall visual score is an additional method for evaluating the degree of lung involvement in a CT scan.

In this study, patients were separated into two groups based on their clinical outcomes: good and worse. Patients whose clinical outcome was intubation and death were assigned to the group with the worse clinical outcome, while the remaining patients were assigned to the group with the good clinical outcome. It should be noted that the ICU physician or the emergency department physician makes the decision regarding intubation for each patient before admission to the ICU. Indications for intubation in this study included severe respiratory distress, loss of consciousness, NIV intolerance, and lack of response to NIV treatment. Patients with poor prognoses, including end-stage cancer, hemodynamic instability despite using vasoactive agents, and critically ill patients, with multimorbidity were not intubated.

Notably, the definition of intubation in this study included patients who have been intubated for at least 24 hours in the ICU.

### 2.3. Statistical Analysis

SPSS software (v. 26) was employed for data analysis. It should be noted that the normal distribution of the data was analyzed via the Kolmogorov-Smirnov test.

Descriptive statistics (mean, variance, standard deviation, and frequency) and inferential statistics included the chi-square test, Student's *t*-test, and multivariate binary logistic regression analysis with a 95% confidence interval (CI). The chi-squared and *t*-test assessed the relationship between dichotomous and quantitative variables and clinical outcomes, respectively. Multivariate logistic regression was used to examine the association between death and independent variables, including age, NLR, comorbidities, oxygen saturation, ICU length of stay, hospital length of stay, and score of CT. Notably, a *P* < 0.05 was considered statistically significant.

## 3. Results

In this study, over 18 months (March 20, 2020, to September 1, 2021), 391 patients with positive PCR were analyzed. The study participants' average age was 63.59 ± 17.76 years, and 57.3% were male. The most prevalent underlying illnesses in this study were HTN (162 patients, 41.4%), DM (156 patients, 39.9%), IHD (82 patients, 21%), and CKD (81 patients, 20.7%). The mean O_2_ saturation was 77.82% ± 11.55 for patients upon entering the ICU.

On the first day of admission, patients' average WBC and NLR were 9.27 ± 5.58 and 10.39 ± 8.66, respectively. There were 44 cases (11.26%) with less than 4000 and 128 cases (32.73%) with a WBC of more than 10000. The mean lung involvement score on HRCT imaging was 14.03 ± 6.04, with alveolar consolidation being the most common type of lung involvement in 133 patients (34%), followed by GGO in 100 individuals (25.6%).  Table 1 presents demographic, clinical, and imaging data from the study's inception.

In the ICU, 152 (38.9%) of the hospitalized patients were intubated, and 149 (38.1%) died. In this study, 16 (4.1%) of the hospitalized patients were intubated but survived, 13 (3.3%) patients were not intubated and died, and 136 (34.8%) patients were intubated and died.

Secondary infection, upper gastrointestinal (GI) bleeding, and venous thrombosis were the most commonly observed complications in patients, occurring in 54 (13.8%), 27 (6.9%), and 17(4.3%) cases, respectively. The patients' average duration of stay in the hospital was 12.87 ± 9.37 days and 10.93 ± 9.44 days in the ICU. At the time of discharge, the mean O_2_ saturation was 92.66 ± 3.04.  Table 2 contains further information on patients' discharge and complications.

In this study, patients were separated into two groups based on their clinical outcomes: good and worse. A total of 165 patients with the outcome of intubation and death (42.2%) were placed in the group with the worse clinical outcome (16 patients: intubated but survived, 13 patients: not intubated and died, 136 patients: intubated and died), while the remaining 226 (57.8%) were placed in the group with the good clinical outcome. The chi-square test and *t*-test were used to determine the relationship between dichotomous variables and quantitative variables and outcomes. Age, DM, HTN, dyslipidemia, CKD, CVA, cerebral hemorrhage, and cancer were reported to be significantly different between these two groups of patients, indicating an increase in the rate of intubation and mortality among these patients. In addition, NLR, the percentage of lung involvement, O_2_ saturation at the ICU and hospital admission, and ICU length of stay significantly differed between the two groups. The results are shown in Table 3.

The relationship between primary variables and clinical outcomes in patients hospitalized in ICU was analyzed with the univariate logistic regression analysis, which confirmed the significant variables in Table 3. The results are shown in the supplementary table (available here).

Multivariate logistic regression analysis based on primary factors (clinical, demographic, imaging, and laboratory) was performed to accurately identify factors affecting ICU patient mortality. The Hosmer-Lemeshow test (*P*=0.785) demonstrated the model's validity. The significant variables, including age, DM, HTN, HLP, CVA, CKD, brain hemorrhage, cancer, primary O_2_ saturation, duration of stay in hospital and ICU, NLR, and score of lung involvement, added to the model at baseline. The final step of the model revealed that the variables DM, HTN, CKD, CVA, brain hemorrhage, NLR index, initial O_2_ saturation, and CT scan score influence the mortality of COVID-19 patients (Table 4).

## 4. Discussion

This study investigated the clinical features and risk variables linked with clinical outcomes in ICU-admitted COVID-19 patients.

According to the results, most of the patients infected with COVID-19 were male (57.30%), which is consistent with the results of previous studies [1, 18, 20, 21]. Some studies have shown more male mortality than female deaths, indicating that viral disorders of the respiratory system are more severe in males and lead to more mortality in men [1, 22, 23]. Our investigation of the link between gender and death revealed no significant correlation. The difference in the study population in terms of the severity of COVID-19, and on the other hand, other factors affecting the mortality of COVID-19 patients, such as concurrent diseases, high levels of inflammatory factors such as interleukin-6 (IL-6), can be the reason for the difference between the results of this study and other studies [24, 25].

Wang et al. [21] showed that COVID-19 patients hospitalized in intensive care units had more comorbidities than patients admitted to non-ICU wards. The present study identified the most prevalent underlying illnesses in people with HTN, DM, and cardiovascular disease. As reported in earlier investigations, DM, hypertension, and heart and renal disease were the most common diseases among COVID-19 patients admitted to the ICU [8, 21, 26]. Hypertension and diabetes in the context of metabolic syndrome are considered to cause a hyper-inflammatory state, leading to a cytokine storm associated with the severity of COVID-19 [27].

The common pulmonary involvement was alveolar infiltration in the form of alveolar consolidation (CONS), GGO, or a combination of both (GGO and CONS), found in 70.85% of patients. In other investigations, GGO and bilateral alveolar involvement have been recorded between 14 and 98% in patients' CT scans [3, 4, 21, 28], indicating the most common type of lung involvement in CT scans of COVID-19 patients. In the current investigation, lung involvement in nodular, reticular, crazy paving, and pleural effusion patterns was detected in fewer patients on CT images.

The mortality rate in the current study was 38.1%, whereas it ranged from 30.9% to 97.0% in previous studies [2, 4, 5], making it higher than the mortality rate for other infectious diseases [2]. COVID-19 lengthens the patient's stay in ICUs and increases patient mortality rates.

Patients admitted to the ICU may require respiratory assistance due to severe hypoxemic respiratory failure. In the current study, 38.9% of hospitalized patients required endotracheal intubation and invasive mechanical ventilation, compared to 15 to 88% reported in other studies [1, 3, 5, 6, 20, 21]. In addition, NIV was used in 43.73% of the patients in the present study, compared to 2.4% to 82.8% in prior studies [3, 21, 29, 30].

Patients (72.17%) who received NIV in the current study required endotracheal intubation, and 89.5% of intubated patients died. In several reports of COVID-19 from Wuhan, China, death rates rose from 52 to 62% among ICU patients and 86 to 97% for those requiring invasive mechanical ventilation [3, 5, 31]. Moreover, early reports of the initial COVID-19 outbreak in the United States indicated that 50–67% of ICU patients and 71–75% of those undergoing invasive mechanical ventilation died [2, 4, 6]. The results of these studies are consistent with the current investigation and suggest that patients who need respiratory assistance with COVID-19 have a higher mortality risk and a shorter survival rate [2].

Based on the high mortality rate among intubated patients, early treatment in the initial and acute phases of the disease with accessible medications should be addressed to avoid the development of ARDS and the necessity for invasive mechanical ventilation.

In the statistical analysis performed between the groups with good and worse clinical outcomes, it was determined that increasing age, the presence of underlying illnesses (HTN, DM, HLP, CVA, CKD, and cancer), a high NLR, a higher percentage of lung involvement, and a lower O_2_ saturation on arrival to the ICU were more prevalent in the group with clinical outcomes of death or mechanical ventilation. In this study, the average age of the participants was over 60, which is associated with underlying diseases that may be one of the causes of ICU admission and deteriorating clinical status [2, 32]. Historically, it has been shown that older age is a strong independent predictor of death in severe acute respiratory syndrome (SARS) and Middle East respiratory syndrome (MERS) [33, 34].

The current investigation demonstrated that rising age is related to mortality among COVID-19 patients. Age-related impairments in T and B cell function and overproduction of type 2 cytokines may lead to abnormalities in viral replication control and extended proinflammatory responses, possibly resulting in adverse outcomes [35].

Inflammation following infectious disease has a crucial role in developing several viral pneumonia types, including COVID-19 [36]. Potential indicators for the prognosis of COVID-19 patients include biomarkers that might reflect inflammation and immunological function. The number of peripheral WBC and the NLR are indications of systemic inflammatory response that have been examined extensively as excellent predictors of the outcome of patients with viral pneumonia [37, 38]. The present study demonstrates that a high NLR is an independent predictive biomarker for COVID-19 patients. The findings of a study conducted by Yang indicated that age and NLR might be connected to the severity of infection. In the present study, an increase in NLR results in a 3% rise in mortality risk and death [38].

In addition to epidemiologic factors, comorbidities may significantly determine the severity and prognosis of COVID-19. As a critical regulator of blood pressure, the angiotensin-converting enzyme (ACE) has been identified as the severe acute respiratory syndrome coronavirus (SARS-CoV) binding site, making hypertension the most prevalent comorbidity.

COVID-19 findings show that 11–58% of all patients had DM, and a COVID-19 mortality rate of 8% has been found in diabetic individuals [7, 26, 39]. DM patients had a 14.2% increased probability of ICU admission [26]. The presence of diabetes, hypertension, CVA, and CKD raises mortality risk by 3.84, 2.36, 5.24, and 2.85 times, respectively, in the current study, indicating that these underlying conditions increase the risk of death in COVID-19 patients.

By reducing the initial O_2_ saturation of patients and increasing lung involvement on CT scans, the probability of mortality rises by 0.94 and 1.10 times, respectively. Medical imaging techniques have a significant role in the early detection and treatment of SARS-CoV-2 infection. A CT scan is an essential diagnostic tool for determining the extent of lung involvement in COVID-19 pneumonia [14]. In COVID-19 patients, pulse oximetry may be utilized as a warning indication to identify “silent hypoxemia.” According to studies, the highest score of CT involvement suggests the lowest oxygen saturation [40].

In China, Yang et al. examined the relationship between chest CT scan parameters and the clinical condition of patients. The CT scan results of 102 individuals with COVID-19 infection showed that the overall CT intensity score is considerably more significant in patients with severe COVID-19 infection compared to moderate cases [41].

The CT intensity score may be used to measure lung involvement severity [42]. Moreover, there is a correlation between the severity of lung involvement and the degree of oxygen saturation, such that the greater the severity of lung involvement, the lower the degree of saturation [40, 42, 43]. Additionally, respiratory distress ≥30 per minute should be noted. Clinical severity criteria include arterial blood oxygen saturation at rest ≤93% or partial arterial blood oxygen pressure (PaO_2_)/fraction of inspiration O_2_ (FiO_2_) ≤ 300 mm Hg [43]. The current investigation also shows that individuals with hypoxia had considerably higher CT intensity scores and a clinically meaningful negative association between CT intensity score and oxygen saturation, which is of significant clinical importance.

### 4.1. Study Strengths and Limitations

The significance of the present study lies in the 18-month length of its duration. It is also noteworthy that the study focused on hospitalized COVID-19 patients with severe illness who required ICU care. In addition to their prognosis, the evaluated patients' clinical symptoms and imaging were also determined. The results may be of great value as they aid in managing such patients.

However, this study had several limitations. One of the study's limitations was the emergence of various types of Alpha to Delta variant coronaviruses during the long period of the study. Considering the limited accessibility to recognizing these various types in conventional clinical environments in Iran, it can highly influence the study findings. Moreover, the emergence of new COVID-19 treatments during the study period may have influenced the results at various phases, which were completely uncontrollable.

Another limitation was considering the amount of just two inflammatory factors (i.e., ESR and CRP) as risk factors. As other inflammatory factors (e.g., D-dimer serum level and interleukin) evaluated in the previous study [24, 25] were not considered risk factors for mortality in all patients, they were omitted and assessed in the current study. Also, considering that the present research was related to the first eighteen months of the outbreak of COVID-19 in Iran, and vaccination was not available to the public until a long period of the study, we were not able to evaluate the role of this essential factor in the prognosis of the patient. It should also be noted that in this study, the patients were assessed only during their stay in the ICU, and there was no information about them after being discharged from the ICU or the hospital.

## 5. Conclusion

Based on the present findings, several features of COVID-19 patients, such as underlying diseases, NLR, and the percentage of lung involvement and primary O_2_ saturation, have a substantial influence on the mortality of these patients. Therefore, early detection of these mortality risk factors for COVID-19 patients may improve their prognosis. It appears that early treatment of patients with these risk factors using existing treatment methods can prevent disease progression and severe outcomes.

## Figures and Tables

**Table 1:** Demographics and baseline characteristics of patients.

		Overall (*N* = 391)						
		General data			*N*		%	
Age		≤60 y			156		39.9	
		>60 y			235		60.1	

Sex		Male			224		57.3	
		Female			167		42.7	

O_**2**_Sat, baseline		O_2_ < 88%			316		80.8	
		92% > O_2_ ≥ 88%			59		15.1	
		O_2_ ≥ 92%			16		4.1	

Comorbidities		HTN			162		41.4	
		DM			156		39.9	
		IHD			82		21	
		CKD			81		20.7	
		COPD			54		13.8	
		CVA			40		10.2	
		DLP			38		9.7	
		ESRD			24		6.1	
		Brain hemorrhage			23		5.9	
		Cancer			12		3.1	
		Hypothyroidism			9		2.3	
		Seizure			9		2.3	
		IPF			7		1.8	
		Cirrhosis			5		1.3	
		Trauma			3		0.8	
		Parkinson			3		0.8	
		Rheumatoid arteritis			2		0.5	
		Sickle cell			2		0.5	
		MS			2		0.5	
		LAM			1		0.3	

Imaging result (score of lung involvement) at baseline		Mild (0–8)			81		20.7	
		Moderate (9–16)			168		43	
		Severe (17–25)			142		36.3	

Type of CT involvement		Consolidation			133		34	
		GGO			100		25.6	
		Mix			44		11.25	
		Reticular + Fibrosis			35		8.95	
		PE			33		8.40	
		Nodular			23		5.90	
		Crazy paving			23		5.90	

Laboratory findings		CRP		Negative	78		19.9	
				+	77		19.7	
				++	131		33.5	
				+++	105		26.9	
		WBC		WBC<4000	44		11.26	
				WBC: 4000–10000	219		56.01	
				WBC>10000	128		32.73	
					Mean ± SD			
		WBC			9.27 ± 5.58			
		ESR			46.05 ± 28.81			
		CPK			396.19 ± 678.29			
		LDH			711.39 ± 414.09			
		NLR			10.39 ± 8.66			
		RBC			4.82 ± 0.78			
		AST			71.61 ± 136.86			
		ALT			63.96 ± 128.31			
		ALP			215.08 ± 121.98			
		PLT			203.44 ± 91.81			
		Cr			1.76 ± 1.89			
		BUN			60.98 ± 51.89			

*N*: number; SD: standard deviation; DM: diabetes mellitus; HTN: hypertension; IHD: ischemic heart disease; DLP: dyslipidemia; COPD: chronic obstructive pulmonary disease; CVA: cerebral vascular accident; ESRD: end stage renal disease; IPF: idiopathic pulmonary fibrosis; MS: multiple sclerosis; LAM: lymphangioleiomyomatosis; CRP: C-reactive protein; ESR: erythrocyte sedimentation rate; CPK: creatine phosphokinase; LDH: lactate dehydrogenase; WBC: white blood cells; NLR: neutrophil-lymphocyte ratio; AST: aspartate transaminase; ALT: alanine transaminase; ALP: alkaline phosphatase; O_2_Sat: oxygen saturation; Cons: considerations; GGO: ground-glass opacification; PE: pleural effusion; y: year.

**Table 2:** Outcomes & complications and discharge characteristics of the patients.

	*N*	%
*Outcomes & complications*		
Intubation	152	38.9
NIV	171	43.73
Death	149	38.1
Discharge	242	61.89
Infection	54	13.8
GI bleeding	27	6.9
Thrombosis	17	4.3
Guillain-Barre syndrome	1	0.25

*O*_*2*_ * sat, discharge with supplementary* O_*2*_		
O_2_ < 88%	11	4.55
92% > O_2_ ≥ 88%	116	47.93
O_2_ ≥ 92%	115	47.52

*N*: number; NIV: noninvasive ventilation; O_2_ sat: oxygen saturation.

**Table 3:** Association between demographic and primary clinical data with outcome.

Variable			Outcome		Total	*P* value
			Good clinical outcome	Worse clinical outcome		
Sex, *F* (%)		Male	132 (58.40)	92 (55.76)	224	0.601^*∗*^
		Female	94 (41.60)	73 (44.24)	167	

Age, *F* (%)		≤60	108 (47.78)	48 (29.09)	156	<0.0001^*∗*^
		>60	118 (52.22)	117 (70.91)	235	

Comorbidity, *F* (%)	Diabetes	Yes	58 (25.66)	98 (59.39)	156	<0.0001^*∗*^
	Hypertension		70 (30.98)	92 (55.76)	162	<0.0001^*∗*^
	Ischemic heart disease		41 (18.14)	41 (24.85)	82	0.108^*∗*^
	Dyslipidemia		15 (6.63)	23 (13.97)	38	0.024^*∗*^
	CKD		34 (15.04)	47 (28.48)	81	0.001^*∗*^
	COPD		27 (11.94)	27 (16.36)	54	0.211^*∗*^
	Hypothyroidism		5 (2.22)	4 (2.42)	9	0.573^*∗*^
	Rheumatoid arteritis		1 (0.44)	1 (0.60)	2	0.669^*∗*^
	Brain hemorrhage		1 (0.44)	22 (13.33)	23	<0.0001^*∗*^
	Parkinson		1 (0.44)	2 (1.21)	3	0.422^*∗*^
	Seizure		6 (2.66)	3 (1.82)	9	0.739^*∗*^
	CVA		10 (4.42)	30 (18.19)	40	<0.0001^*∗*^
	Sickle cell		1 (0.44)	1 (0.60)	2	0.667^*∗*^
	ESRD		14 (6.20)	10 (6.06)	24	0.956^*∗*^
	Cancer		2 (0.88)	10 (6.06)	12	0.004^*∗*^
	Trauma		0	3 (1.82)	3	0.074^*∗*^
	LAM		0	1 (0.60)	1	0.422^*∗*^
	MS		1 (0.44)	1 (0.60)	2	0.578^*∗*^
	Cirrhosis		3 (1.32)	2 (1.21)	5	0.645^*∗*^
	IPF		2 (0.88)	5 (3.03)	7	0.117^*∗*^

O_2_ saturation ranges at baseline, *F* (%)		O_2_ < 88%	169 (74.78)	147 (89.09)	316	0.002^*∗*^
		92% > O_2_ ≥ 88%	44 (19.47)	15 (9.09)	59	
		O_2_ ≥ 92%	13 (5.75)	3 (1.82)	16	

The score of lung involvement at baseline, *F* (%)		0–8	50 (22.12)	31 (18.79)	81	0.002^*∗*^
		9–16	110 (48.68)	56 (33.94)	168	
		17–25	66 (22.20)	78 (47.27)	142	

Laboratory findings at baseline	CRP, *F* (%)	−	45 (19.91)	33 (20)	78	0.981^*∗*^
		+	43 (19.02)	34 (20.60)	77	
		++	77 (34.08)	54 (32.73)	131	
		+++	61 (26.99)	44 (26.67)	105	
	ESR	Mean ± SD	44.22 ± 28.21	48.56 ± 29.52	391	0.142^*∗∗*^
	CPK		408.98 ± 710.90	378.68 ± 632.60	391	0.663^*∗∗*^
	LDH		702.28 ± 436.34	723.87 ± 382.47	391	0.611^*∗∗*^
	WBC		8.80 ± 5.45	9.91 ± 5.70	391	0.051^*∗∗*^
	NLR		9.49 ± 8.71	11.62 ± 8.46	391	0.016^*∗∗*^
	BUN		57.23 ± 58.84	66.13 ± 40.08	391	0.094^*∗∗*^
	Cr		1.76 ± 2.25	1.76 ± 1.24	391	0.981^*∗∗*^
	PLT		205.42 ± 96.94	200.72 ± 84.50	391	0.617^*∗∗*^
	AST		75.78 ± 171.04	65.90 ± 65.95	391	0.481^*∗∗*^
	ALT		70.19 ± 160.57	55.42 ± 60.45	391	0.261^*∗∗*^
	ALP		204.62 ± 107.43	229.40 ± 138.54	391	0.047^*∗∗*^

SpO_2_ at baseline		Mean ± SD	80.65 ± 10.32	73.96 ± 12.04	391	<0.0001^*∗∗*^

ICU length of stay		Mean ± SD	9.99 ± 6.33	12.22 ± 12.41	391	0.021^*∗∗*^

Hospital length of stay		Mean ± SD	11.83 ± 6.22	14.31 ± 12.33	391	0.010^*∗∗*^

^*∗*^Chi-square test,^*∗∗*^student's *t*-test.

**Table 4:** Relationship between variables and outcome based on multivariate logistic regression.

Variable	*B*	S.E.	*P* value	Odds ratio (OR)	95% C.I. for OR	
					Lower	Upper
Age^a^	0.294	0.295	0.320	1.34	0.752	2.393
DM	1.346	0.297	<0.0001	3.84	2.145	6.880
HTN	0.862	0.294	0.003	2.36	1.330	4.214
HLP	0.140	0.429	0.744	1.15	0.497	2.665
CVA	1.656	0.465	<0.0001	5.24	2.108	13.025
CKD	1.047	0.339	0.002	2.85	1.468	5.534
Cancer	1.794	0.980	0.067	6.01	0.881	41.059
Brain hemorrhage	3.371	1.125	0.003	29.10	3.207	264.121
O_2_ saturation	−0.062	0.013	<0.0001	0.94	0.917	0.963
Hospital length of stay	−0.019	0.117	0.869	0.98	0.779	1.234
ICU length of stay	0.033	0.116	0.778	1.03	0.822	1.298
NLR	0.044	0.016	0.007	1.045	1.012	1.079
CT score	0.101	0.026	<0.0001	1.10	1.051	1.165
Constant	0.467	1.144	0.683	1.59		

The table shows all the variables: −2loglikelihood = 343.726; *χ*^2^ = 175.981, *P* < 0.0001. Hosmer-Lemeshow statistics = 4.738 with d*f* = 8, *P*=0.785. ^a^: age > 60.

## Data Availability

All data generated or analyzed during this study are included in this published article.

## Supplementary Materials

Supplementary Materials Relationship between variables and outcome based on univariate logistic regression.
