# Supplementary material for: Analyzing Trends in Demographic, Laboratory, Imaging, and Clinical Outcomes of ICU-Hospitalized COVID-19 Patients
Source: Can J Infect Dis Med Microbiol. 2023 May 29;2023:3081660. doi: 10.1155/2023/3081660 (PMC10241583; doi:10.1155/2023/3081660)
Supplement: Supplementary Materials — Relationship between variables and outcome based on univariate logistic regression. [file 3081660.f1.docx]

| ***relation between variables and outcome base on univariate logistic regression*** | | | | | | |
| --- | --- | --- | --- | --- | --- | --- |
| **variable** | **B** | **S.E.** | **p. value** | **Odds ratio (OR)** | 95% C.I.for EXP(B) | |
|  |  |  |  |  | Lower | Upper |
| Age  (>60) | 0.802 | 0.217 | <0.0001 | 2.231 | 1.458 | 3.414 |
| DM | 1.444 | .220 | <0.0001 | 4.237 | 2.754 | 6.519 |
| HTN | 1.033 | 0.213 | <0.0001 | 2.809 | 1.851 | 4.262 |
| HLP | 0.823 | 0.349 | 0.018 | 2.278 | 1.149 | 4.517 |
| CKD | 0.811 | 0.254 | 0.001 | 2.249 | 1.368 | 3.698 |
| CVA | 1.569 | 0.381 | <0.0001 | 4.800 | 2.274 | 10.134 |
| Brain hemorrhage | 3.544 | 1.028 | 0.001 | 34.615 | 4.615 | 259.627 |
| cancer | 1.978 | 0.782 | 0.011 | 7.226 | 1.562 | 33.435 |
| spo2 at baseline | -0.054 | 0.010 | <0.0001 | 0.948 | 0.929 | 0.966 |
| hospital length of stay | 0.030 | 0.012 | .015 | 1.030 | 1.006 | 1.056 |
| ICU length of stay | 0.026 | 0.012 | .028 | 1.026 | 1.003 | 1.050 |
| Score of lung involvement at baseline | .064 | .018 | <0.0001 | 1.066 | 1.029 | 1.103 |
| NLR | 0.029 | 0.012 | 0.019 | 1.029 | 1.005 | 1.054 |
